# Exploring the Potential Impact of Training on Short-Term Quality of Life and Stress of Parents of Children with Autism: The Integrative Parents’ Autism Training Module

**DOI:** 10.3390/ijerph21040474

**Published:** 2024-04-13

**Authors:** Nikoletta Mavroeidi, Christos Sifnaios, Ariadne Ntinou, Giorgos Iatrou, Olympia Konstantakopoulou, María Merino Martínez, Martina Nucifora, Ibrahim Tanrikulu, Antonios Vadolas

**Affiliations:** 1 Department of Scientific Documentation and Education, Child and Adolescent Center, 12123 Peristeri, Greece; sifneos@kpechios.org (C.S.); giatrou@kpechios.org (G.I.); avadolas@kpechios.org (A.V.); 2 EPIONI Greek Carers Network, 15125 Marousi, Greece; infoepioni@gmail.com; 3 Department of Psychology, University of West Macedonia, 53100 Florina, Greece; 4 Center for Health Services Management and Evaluation, Department of Nursing, National and Kapodistrian University of Athens, 11527 Athens, Greece; olykonstant@nurs.uoa.gr; 5 Asociación Autismo Burgos, 09001 Burgos, Spain; psicologia@autismoburgos.org; 6 Controvento Onlus, 95123 Catania, Italy; martinanucifor@hotmail.it; 7 Department of Psychological Counseling & Guidance, Faculty of Education Gaziantep University, Şehitkamil-Gaziantep 27310, Turkey; ibrahimtanrikulu@gmail.com

**Keywords:** ASD, autism, parents, training, psychoeducation, stress, quality of life

## Abstract

Parents of autistic children experience high levels of parental stress and low quality of life related to the demanding child caring burden they experience. Parent education and training programs are acknowledged to improve parental well-being and reduce parenting stress. In the framework of the Erasmus+ Integrative Autism Parents Training Project (IPAT), we developed the IPAT Training Module based on parents’ expressed needs, in order to improve parental quality of life (QoL) and decrease their perceived stress. Sixty-two parents from four countries participated in the IPAT Module Training activity. We used WHOQOL-BREF and Perceived Stress Scale (PSS-10 version) for QoL and stress, respectively, before and after training and a study-specific questionnaire to assess participants’ satisfaction. Parents’ QoL improved significantly in the environment domain and specific items, while stress levels remained unmodified. Training appeared more advantageous for parents with lower initial QoL and those whose child had been enrolled in a special education program for an extended duration. Parents were quite satisfied, in particular those with lower initial social relationships QoL. Larger studies including a control group are necessary to support preliminary evidence provided by this study, identify additional effect moderators, and disentangle the contribution of different components of the training.

## 1. Introduction

Autism or Autism Spectrum Disorders (ASD) are neurodevelopmental conditions with a wide range of presentations, needs, abilities, and challenges. Autistic individuals present difficulties concerning verbal and non-verbal social communication and interaction, as well as restrictive or repetitive interests, behaviors, or movements [1,2]. The severity of the challenges encountered and the level of needs may vary substantially, ranging from individuals who require only limited support to those with restricted autonomy [3,4,5].

The role of the family is pivotal for meeting the needs of autistic people in a comprehensive and assiduous manner; parents constitute the main supportive and continuous care provision system, while efficient collaboration with the health and education professionals caring for their child is essential [6]. Raising an autistic child may be stressful for parents and for the whole family. Parents of a child with ASD will go through different stages in their life: pre-diagnosis, diagnosis, family life adjustment, navigating the available services and supports, parental empowerment, and moving forward [7]. After receiving a diagnosis, parents often undergo a strenuous process of accepting that their child has autism. At the same time, they seek information about the condition, they need to choose between different interventions available, advocate for their child’s rights, and access appropriate services [8]. Accessing appropriate and reliable information about autism and available support is equally challenging, with considerable impact on their child and themselves [9].

The parenting of autistic children is being shown to pose more challenges than the parenting of neurotypical children or children with other disabilities, for example, Down syndrome [10,11]. More specifically, parents of children with autism experience higher levels of parental stress and psychological distress, with discrepancies in the psychological impact reported between mothers and fathers, as compared to parents of neurotypical children. Parenting stress is characterized by distress that arises from the demands faced within the parental role [12]. Behavioral issues of their children and the severity of ASD symptoms are among the major contributors to the stress of parents of autistic children [13,14]. Autistic people’s social and behavioral issues, often leading to alienation, stigmatization, and bullying, are potentially associated with depression and anxiety or aggravating behavioral challenges which may exacerbate parental stress [15]. Limited resources, inadequate professional guidance, and the stigma associated with ASD have also been reported to cause concern and increase parental stress [9]. Parents of autistic persons who perceived themselves to be resilient reported lower levels of parental stress [11]. In addition to the psychological and physical hardships, financial challenges constitute another important stress factor; parents of autistic children experience a higher frequency of work loss and additional costs for ensuring the necessary medical or other services needed for their child [16,17,18]. Over the last 15 years, the socioeconomic status of the parents has been found to be associated to some extent with parental and family stress [19]. Effective coping strategies include creating a strong social support system, which enhances resilience and improves well-being, and redefining and restructuring the experience of raising a child with autism, so it is conceived as an advantageous experience [20].

One measurement for well-being frequently used is quality of life (QoL) [21]. The World Health Organization defines quality of life as an “Individuals’ perceptions of their position in life in the context of the culture and value systems in which they live and in relation to their goals, expectations, standards, and concerns” [22] (p. 551). The level of QoL is linked to the resources available and issues of self-determination, purpose in life, and sense of belonging [16,23]; its concept is broad and encompasses a combination of physical and psychological health, personal independence and relationships, as well as firmly held views and judgments, along with the way all of these affect the individual’s interaction with their environment [20]. Caring for a child with ASD lowers the parents’ quality of life, as compared to parents of typically developing children or to parents of children with other disabilities [24]. The possible manifestation of behaviors that cause concern in autistic individuals has been reported to negatively impact their parents’ QoL [10]. Quality of life can be improved through specific coping strategies that can help parents deal with stressful situations [25]. Therefore, it is essential to create and enhance intervention and support services for parents of autistic children in order to improve their quality of life [26]. Furthermore, their quality of life is worth investigating, since it is pivotal in developing improved interventions and support services to aid parents in maintaining both their physical and mental health and thus, in caring adequately for their child [20].

Parents of children with ASD need to have support and training to deal with the challenges they experience. Parental training is one of the factors that allows optimal adaptation of parents to their child’s autism diagnosis, as well as their ability to support treatment and collaborate constructively with the respective professionals. Parental training is known to provide knowledge and a better understanding of the disorder, enhance parental skills, decrease parental stress, and improve their self-perceived quality of life [27,28]. This positive impact is not only viewed in training delivered in conventional educational settings but also in those employing modern audio-visual technology, which started with the use of VHS and DVD and expanded widely with the widespread use of the Internet [29,30,31]. The participation of parents in training seems to positively impact their children in making gains in language, communication, and socialization, improving child–family interaction, reducing parental stress, and increasing self-efficacy [32,33,34,35]. Nevertheless, results may be contradictory, as there have been observations of no significant impact upon stress in particular types of training, such as psychoeducation [36]; increases in stress after a certain time has passed [37]; persistence of low self-competence; and high stress linked to negative life events [13].

One of the main problems, which could explain some of the conflicting findings, is the definition of training. Different studies produce contradictory results because they do not describe the same thing; ‘parents’ training’ is not an area that is clear, unequivocal, or undisputed, with a variety of terminology used in the literature [38]. The terms “education” and “training” have been used in the past either as opposite or interchangeable terms, leading to uncertainty and difficulty in reviewing the relevant literature [34,39]; the variety in formats, intensity, location, duration, and target age groups further adds to the complexity and need for clarification [40]. However, two main categories refer to, on the one hand, programs that offer parent-focused support and knowledge (mostly described under the term ‘education’) and, on the other, programs that provide parents with child-focused skills and techniques as a means for parent-mediated interventions, mostly described under the term ‘training’. The first category includes formats such as care coordination and psychoeducation, whereas the second includes primary and complementary parent-mediated interventions for core symptoms and disruptive behaviors or behaviors that cause concern [40]. It seems, however, that successful educational programs, whatever we call them, support parents both in understanding their children’s behaviors and managing them [41].

Psychoeducation combines information and therapeutic elements to help patients, family members, and clinicians manage illness or disability more effectively [27,42]. It can be viewed as a specialized form of education aimed at helping people learn and increase awareness about a range of emotional and behavioral difficulties and their effects and develop strategies for dealing with them. In autism care, the aim of psychoeducation is to inform parents of autistic children about the disorder, including evidence-based treatments, to help them adapt to difficulties and improve their problem-solving skills [43]. Acquiring knowledge (information about ASD and interventions), developing skills (coping with behaviors that cause concern, communication, problem solving), and providing support (group support, shared experiences, social networking) constitute aspects of psychoeducation, being a combination of all three in most cases [25,40].

Evidence shows that psychoeducation interventions constitute a valuable resource for improving the quality of life (QoL) of parents of autistic persons. Musetti et al. [44] carried out a large review of studies on the quality of life of parents with a child or adolescent with ASD and their findings indicated that direct parental involvement in psychoeducation programs was associated with increased parental quality of life. Although they do not directly address parental quality of life and stress, other studies document the positive impact of psychoeducation programs on factors affecting parental stress and quality of life. Ericson et al. [20] designed a psychoeducational group intervention aimed at supporting adolescents with mild intellectual disability and their parents and assessed its effectiveness. The program was named “The Super Control” project and focused on helping parents and their adolescent children develop self-control and self-regulation skills. The results suggested that participating in the program led to positive outcomes with regard to participants’ understanding of the diagnosis, dealing with everyday difficulties and social networking. The project had a positive impact on parental awareness of how parents can become more proactive in dealing with the future, gaining more understanding of autism, and seeking appropriate interventions [20]. In another interventional research study, DaWalt et al. [45] developed and implemented a multi-family group psychoeducation program called “Transitioning Together”, aiming at providing support and information to adolescents with ASD and their parents during the transition period from adolescence to adulthood. Different aspects of family functioning, mental health, and self-determination for both the adolescents with ASD and their parents were assessed. The results indicated that families who participated in the Transitioning Together program showed improvements in various areas, including increased family cohesion, reduced family conflict, improved mental health outcomes, and enhanced self-determination skills for both adolescents with ASD and their parents.

Considering the positive impact that psychoeducation has on the lives of parents of autistic children, a psychoeducational training module for parents of autistic children was designed as part of the Erasmus+ co-funded project “Integrative Parents’ Autism Training” (IPAT) that lasted from November 2020 to May 2023. This was a collaborative effort between five organizations and institutions in Greece, Spain, Turkey, and Italy. The IPAT training module was developed based on the results of a previous research study involving four focus groups of parents of children with ASD in which they expressed their needs. Analysis of the results revealed a number of different parental needs that formed the basic framework for developing the content of the IPAT training module (www.ipatproject.eu, accessed on 10 December 2023). The aim of our study was to preliminarily explore the potential efficacy of psychoeducation using the IPAT module on improving parental self-perceived quality of life and reducing stress and to assess participants’ satisfaction with the module and training, as well as identify potential disparities across countries. More specifically, our primary research hypothesis was that QoL would improve and parental stress would decrease after training. The secondary objective of our study was to identify factors influencing parental QoL and stress, potential efficacy of the intervention, and overall satisfaction with the module and training.

## 2. Methods

### 2.1. The Intervention: IPAT Training Activity Using the IPAT Module

The IPAT training module was structured in a way that balances academic content and accessible, applicable, skill-building knowledge, based on the parents’ needs, as expressed by them in 4 focus groups at the beginning of the project. Following an extensive analysis of the focus group outcomes, a total of eight prominent themes surfaced, aligning closely with the subject matter addressed during the IPAT training sessions. This process unfolded across a span of six months leading up to the commencement of the IPAT training, during which a group of experienced professionals specializing in autism and mental health, encompassing both clinical practitioners and academics, meticulously crafted the structure and content of each presentation. Furthermore, the finalized material underwent rigorous scrutiny by an external collaborator possessing substantial expertise in autism research and clinical practice.

The intervention was delivered in groups and took place in 8 meetings lasting approximately 3 h each, from March to June 2022. Meetings were held either in person (in Italy and Turkey) or online (in Greece and Spain) and delivered in the native language of each country. There were 8 key topics that the IPAT module focused on: “What is autism”, Therapeutic approaches”, ”Lived experience of the family”, “Child–Parent interaction”, “Social inclusion and autonomy”, “Adolescence and adult life”, “Legal issues and rights”, “Practical guidance and useful tools—Prevention and management of accidents and health issues”. It consisted of 31 short PowerPoint presentations (15 min each), followed by an unstructured discussion of each presentation topic, and 7 structured interactive activities (45–60 min each) corresponding to the main topic of the first 7 meetings. Instead of an interactive activity, in the 8th meeting, parents reflected on the experience of their participation in the IPAT training. This training material was available in English, Spanish, Italian, Greek, and Turkish. In several presentations, modifications were made to specific elements of the content to ensure that it resonated more deeply with the cultural background and expectations of the participants in each respective country. These adjustments included tailoring information to reflect the specific available services, advocacy organizations, and pertinent legal and regulatory frameworks within each country.

The sessions of each meeting were delivered and coordinated by two experienced mental health professionals in each country, with group discussion, exchange of ideas, and experience sharing among participants being an essential feature of all sessions. All participants signed a confidentiality agreement via email before the start of the training intervention. The intervention was offered at no cost to participants. There were two training meetings organized for the coordinators of all countries and guidelines for coordinators were available. Each session of the IPAT training was meticulously structured and described, providing identical guidelines to the coordinators for the delivery of content and interactive activities. Subsequent discussions after each presentation implemented an open and unstructured format, encouraging participants to pose queries and engage freely with the content presented.

### 2.2. Participants

Participants of the present study were parents of autistic individuals from Greece, Italy, Spain, and Turkey, recruited in the respective countries. All participants had been invited to participate voluntarily in the IPAT psychoeducation program and freely complete the scales for the self-perceived quality of life and perceived stress pre- and post-training, as well as the satisfaction survey at the end of the program.

There were no constraints or exclusion criteria as to the age of the autistic child and the level of their needs or degree of autonomy, in the four partner countries. All participants met the following selection criteria. (a) They had no prior experience of attending a psychoeducation or other training program in autism; (b) they actively committed to the IPAT training activity until its completion; (c) they were fluent speakers of the language in which the IPAT training was delivered; and (d) there was no diagnosis of any intellectual disability. No criteria based on socioeconomic status, level of education, or other demographic factors were used to exclude participants.

Participants in Greece and Spain were recruited through the creation of an invitation poster detailing the requirements for participation in the IPAT training activity. The invitation was distributed to parents of autistic children through professionals and organizations in Greece and Spain, such as regional and nationwide institutions and day centers for autism, family associations, private practitioners and professionals, and schools. In Turkey and Italy, parents were given hard copies of the invitation to participate in the IPAT training in person through the local services they attended. Overall, 62 participants took part in the IPAT training module, 18 in Greece, 18 in Spain, 11 in Italy, and 15 in Turkey.

### 2.3. Study Design

We conducted a pre- and post-training single-group assessment and a satisfaction survey in order to explore the potential benefits of the IPAT psychoeducation intervention on parental quality of life and stress and assess the participants’ satisfaction with the IPAT training and module.

### 2.4. Measures

#### 2.4.1. Quality of Life

The quality of life of parents participating in the IPAT module was measured using the WHOQOL-BREF questionnaire. The WHOQOL-BREF questionnaire assesses the quality of life, focusing on an individual’s standards, personal goals, concerns, value systems, and culture. It is a 26-item instrument consisting of four domains: physical health (7 items), psychological health (6 items), social relationships (3 items), and environmental health (8 items). There are also two questions that are examined separately (question 1 ≥ individual’s overall perception of quality of life, question 2 ≥ individual’s overall perception of their health [22]). We selected this questionnaire because it had been standardized in each partner country of the IPAT program, it is widely used, and it is suitable for use in multiple cultural and national contexts, allowing for comparison across countries [22]. Its length is one-quarter of the WHOQOL-100, but it incorporates good breadth and comprehensiveness with the inclusion of items from each of the 24 facets of quality of life included in the longer form. Further, the WHOQOL-BREF uses a subset of items included in the longer version, which allows direct comparison between data collected from specific populations using either of the two assessments.

Each individual item is scored on a 1 to 5 Likert scale. The domain-specific average scores denote an individual’s perception of quality of life in each particular domain. Domain scores are scaled in a positive direction (i.e., higher scores denote higher quality of life). The mean score of items within each domain is used to calculate the domain score. The measure is calculated by summing the point values for the questions corresponding to each domain and then transforming the scores to a 20–100-point interval; mean scores are multiplied by 4 to make domain scores comparable with the scores used in the WHOQOL-100. The first two questions of the WHOQOL-BREF do not correspond to a domain, but mean scores of each question are also calculated as described above.

#### 2.4.2. Stress

For measuring parents’ stress, the Perceived Stress Scale (PSS-10 version) was used [46]. There are three versions of this questionnaire. The original instrument (PSS-14) was developed in English and it was subsequently shortened to 10 items (PSS-10) with the usage of factor analysis based on data from a sample of 2387 U.S. residents. A four-item PSS (PSS-4) was introduced by Cohen and Williamson [47], but later studies questioned its psychometric properties [48,49]. The selection of this research tool for our research was also based on the fact that it is standardized in each partner country of the IPAT program and it is widely used. Parents rated each item on a five-point Likert scale from ‘never’ to ‘very often’ (0 = never, 1 = almost never, 2 = sometimes, 3 = fairly often, 4 = very often). To calculate a total PSS score, responses to the four positively stated items (items 4, 5, 7, and 8) first need to be reversed (i.e., 0 ≥ 4; 1 ≥ 3; 2 ≥ 2; 3 ≥ 1; 4 ≥ 0). The PSS score is then obtained by summing the points awarded to the 10 items and ranges from 0 to 40 as follows: 0–13 = Low stress, 14–26 = Moderate stress, 27–40 = High perceived stress.

#### 2.4.3. Satisfaction with the IPAT Training Module

We also developed a study-specific self-administrated questionnaire for the satisfaction survey that consists of nine questions. Six of them, rated from 1 = worst to 10 = excellent, are used to rate satisfaction with specific aspects of the training and module: the training overall, presentations, interactive activities, moderators, time for discussion and interaction, fulfillment of expectations. The remaining three questions were open-ended, in which participants were requested to provide text answers about 1. additional topics they would have liked to address that had not been considered in the IPAT module, 2. suggestions for improvement of the module and training, 3. additional comments about their participation in the training activity.

#### 2.4.4. Demographics, ASD-Related, Service Use and Attendance Information Form

For the first administration, 19 initial questions were used in order to collect socio-demographic, ASD-related, and service use data for the participants (age, gender, education level, marital and employment status, rural/urban place of residence, previous/current attendance of counselling/psychotherapy programs) and their children (age, gender, diagnosis, time from delivery of the diagnosis, level of needs, ability to speak fluently, medication in order to control any behaviors that cause concern, participation in rehabilitation program, enrollment in a special education program). In order to record the diagnosis delivered to their child, parents were requested to select the respective diagnosis from a list of clinical terms coded with ICD-10, DSM-IV-TR, and DSM-5, included in this information form.

For the second administration, 2 initial questions were used referring only to the participants: number of meetings of the IPAT psychoeducation program they had attended and the reason they stopped before completing the program, in case they had done so.

### 2.5. Data Collection

Participants received the questionnaires via email in a Google forms link, in the case of participants from Greece and Spain, and a hard copy for the participants from Italy and Turkey. There were two administrations of the questionnaire, the first one just before the first meeting of the start of the IPAT training module, and the second one two weeks after the last meeting. Each participant received a unique anonymous code in the first administration of the measures, in order to protect the privacy and confidentiality of the data used for analysis.

Data collection as per the satisfaction survey was conducted using a similar procedure, after completing the second administration of the WHOQOL-BREF and PSS-10 questionnaires.

### 2.6. Statistical Analysis

Categorical variables are presented as absolute (n) and relative (%) frequencies, while quantitative variables are presented as mean (standard deviation) or median (interquartile range). The Kolmogorov–Smirnov test and normality plots were used to test for normal distribution of quantitative variables.

In order to test our primary study hypothesis, we used paired samples *t*-test and analysis of variance for repeated measures, to compare pre- and post-training domain-specific mean QoL and perceived stress scores, in total and within countries, as well as across countries, respectively. In the analysis of variance for repeated measures, the Mauchly test of sphericity was applied to investigate the presence of circular shape symmetry, in which case the sphericity test was applied, whereas the Greenhouse–Geisser test was applied in the absence of circular symmetry. In order to identify factors influencing parental QoL and stress and potential efficacy of intervention, we conducted bivariate and multivariate analysis to explore correlations between the dependent variables and independent ones. Dependent variables were (a) baseline QoL domain-specific and stress scores, and (b) participants whose score improved post-training and participants whose score did not improve, as a new dichotomous dependent variable that we created. We defined participants whose score improved as those with a higher respective domain QoL score post-training or with reduced total perceived stress score to a lower stress level; we calculated the respective proportions of participants and compared those with improved score to those without, with respect to the independent variables. Independent variables were country, other socio-demographics, ASD- and service use-related characteristics, number of meetings attended, and participants’ overall satisfaction with the training.

The baseline domain-specific QoL and stress scores were also considered in the independent variables when exploring factors influencing potential efficacy of intervention, as described above.

The chi-square test, student’s *t*-test, the Mann–Whitney test, and Pearson’s and Spearman’s correlation coefficients were employed, as appropriate for count data, in the bivariate analysis.

We conducted multivariate analysis, including independent variables identified to be significantly correlated with the dependent variables at the level of 0.20 (*p* ≤ 0.20). Multivariate linear regression was applied (backward stepwise linear regression), and coefficients’ beta, or odds ratios for dichotomous dependent variables, and the corresponding 95% confidence intervals and *p*-values, were calculated.

The two-sided level of statistical significance was set equal to 0.05. Data analysis was conducted using the Statistical Package for Social Sciences IBM SPSS 22.0.

### 2.7. Ethics

The questionnaires were completed anonymously after written informed consent was provided by participants. Data were coded and non-identification was further enhanced by the collection procedure. After collection, the data were made accessible only to selected members of the IPAT research team and were used exclusively for scientific purposes, within the aim and objectives of the study. Participants had been informed in writing about all the above. Furthermore, they had been informed that their participation or lack thereof should be a decision of free will and the participation in the survey would be independent of their participation in the training activity or any other service provided to their child or themselves. The implementation of the study was approved by the Hellenic National Agency Erasmus Plus, Reference number 13770/23-7-20.

## 3. Results

Overall, 62 parents of autistic children from four countries, Greece, Italy, Spain, and Turkey, participated in the IPAT training activity using the IPAT module, and response varied as follows: 84–89% across WHOQOL-BREF questionnaire domains, 90% for PSS-10, and 76% for the satisfaction survey. Sixty-one parents reported on their gender: fifty-three females (86.9%) and eight males (13.1%), with a mean age of 42.8 years. Forty-eight of their children (77.4%) were males and fourteen (22.6%) were females, with a mean age of 9.2 years. Sixty-one parents reported about their place of residence; fifty-four (88.5%) live in an urban area, while seven (11.5%) live in a rural area. In total, 16 parents (26.2%) had previously attended or were attending a counseling or psychotherapy program at the time of the study. Fifty-six parents reported on the number of meetings they had attended; nine parents (16.4%) had participated in up to three meetings, seventeen (30.9%) in four to six meetings, and twenty-nine (52.7%) in seven to eight meetings, the average number of meetings attended being 5.9 (SD = 2.2), the median 7 (IR = 3). The main reason reported for quitting before completing the eight meetings was “lack of time or other responsibilities” (34%) and “health issues” (19.4%). Main demographics and characteristics of the study participants and their children are presented in Table 1.

More than half of the children had been diagnosed with Childhood Autism (58.1%) and one out of five with Asperger Syndrome (21%); each one of the remaining diagnoses was delivered to less than 10% of the children. Mean time since diagnosis was 6.4 years (SD 4.2). Fifty-seven parents reported their child’s level of needs, with 23–40.4% rating them as low, 22–38.6% as medium, and 12–21% as high. Participants differed across countries with respect to the age of both the parents and the child, the distribution of diagnoses, years since diagnosis, and number of meetings they attended, as shown in Table 1.

In addition, half of the children were described by their parents as “able to speak fluently”. Almost one-third of the children (29%) were receiving medication to control behaviors of concern at the time of the study. Four out of five children (80.6%) attended a rehabilitation program (at a day center or other group or individual programs), for 5.7 h per week and for 4.3 years in total on average, respectively; almost half of the children (43.5%) were enrolled in a special education program (special school, inclusion class, personal assistance) for an average of 4.8 years.

Mean scores for quality of life and perceived stress of parents by administration and country are presented in Table 2.

Baseline QoL ranged from 56 in the environment domain to 62.8 in the physical health domain, the difference being non-significant across the different domains (*p* > 0.05).

We have identified the following factors interfering with baseline QoL and stress; results presented refer to multivariate regression analyses. Participants from Turkey had a lower QoL physical health domain initial score (*p* = 0.048; R^2^ = 0.812, *p* < 0.001). Low level of needs and higher number of years their child was involved in a special education program were both found to be independently related with higher QoL psychological domain initial score (*p* = 0.021 and *p* = 0.025, respectively; R^2^ = 0.276, *p* < 0.001). Higher overall satisfaction with the IPAT training program and parental age were both independently related to lower QoL social relationships domain initial score (*p* = 0.002 and *p* = 0.034, respectively; R^2^ = 0.271, *p* < 0.001). Participants from Spain and those residing in urban areas had a higher QoL environment domain initial score (*p* = 0.005 and *p* = 0.030, respectively). In contrast, participation in four to six meetings and higher initial perceived stress score were related to lower QoL environment domain initial score (*p* = 0.013 and *p* = 0.028, respectively) (R^2^ = 0.37, *p* < 0.001). Finally, unemployed/homemaker participants and parents of children with “autism” diagnosis were found to be more stressed pre-training, as they had a higher initial total perceived stress score (*p* = 0.039 and *p* = 0.045, respectively), while participants from Italy were initially less stressed than those from other countries (R^2^ = 0.315, *p* < 0.001).

We obtained the following results with respect to our primary study hypothesis on QoL improvement and reduction in stress post-training. Quality of life of participants improved significantly post-training with respect to the QoL environment domain mean score (*p* = 0.011). The physical health, psychological and social relationships QoL domain scores were not modified significantly post-training, (*p* = 0.264, *p* = 0.984, and *p* = 0.726, respectively). Total perceived stress was not modified significantly post-training, being initially at a moderate level on average (*p* = 0.453).

Within countries, only participants from Turkey presented with significantly improved QoL scores in all four domains post-training, (*p* = 0.001, *p* = 0.081, *p* = 0.021, *p* = 0.002, respectively), whereas their total stress score had significantly increased (*p* = 0.048), remaining, however, at the same moderate level. No significant modification of the QoL nor stress scores was identified in participants from Greece, Italy, and Spain, as presented in Table 2.

According to the analysis of variance for repeated measures, no statistically significant association was found between time of administration and country and any of the QoL domains nor total stress scores post-training, (*p* = 0.717, *p* = 0.976, *p* = 0.919, *p* = 0.812, and *p* = 0.527, respectively). Similarly, no statistically significant relationships were found with parents’ or child demographics, ASD-related or service use characteristics (*p* > 0.05).

Within domains, quality of life improved significantly with respect to specific items:

Environment QoL_9: “How healthy is your physical environment?” (*p* = 0.010), QoL_14: “To what extent do you have the opportunity for leisure activities?” (*p* = 0.031), QoL_24: “How satisfied are you with your access to health services?” (*p* = 0.015), Psychological domain QoL_5: “How much do you enjoy life?” (*p* = 0.034), Physical health domain QoL_16: “How satisfied are you with your sleep?” (*p* = 0.023).

Participants with improved QoL scores or less stress post-training are presented in Table 3.

We have identified the following factors correlated with potential efficacy of the intervention; results presented refer to multivariate regression analyses.

Participants with improved QoL physical domain score post-training were more likely to have had lower QoL physical domain initial score (95% CI 1.018–1.518, *p* = 0.033; R^2^ = 0.812, *p* < 0.001). Participants with improved QoL psychological domain score between the two administrations were more likely to have a higher number of years spent by their child in a special education program (95% CI 1.020–11.565, *p* = 0.033; R2 = 0.724, *p* < 0.001). No significant associations were found between improved QoL social relationships and environment domain scores, nor for reduced total perceived stress, with any of the dependent variables (*p* > 0.05).

Participants’ satisfaction with specific aspects of the IPAT training using the IPAT module is presented in Table 4. Mean satisfaction by item ranged from 8.4 to 9 (fulfillment of expectations and satisfaction with moderators of the training activity, respectively) (median = 9, IQR = 2).

One factor was identified in the multivariate analysis to be independently correlated with overall satisfaction with the training; participants who attended or were currently attending a professional counselling or psychotherapy program were overall less satisfied with the IPAT psychoeducation program than those who did not (*p* = 0.039; R^2^ = 0.252, *p* < 0.001).

In total, eight participants (13%) answered positively on additional topics they would have liked to be included in the training, six of whom (75%) stated that they would have liked more practical examples on specific issues to be included.

Twenty one participants (34%) formulated suggestions for improvement of the training; six of them (28.5%) suggested organization- and delivery-related improvements (a follow-up session, a more specific preparation session, a final meeting of parents with their children, and the organization of an online platform to communicate with each other or with special therapists), 7–33.3% suggested longer (3) or shorter (3) duration of the training meetings, while 4–19% perceived improvement in terms of additional information to be provided on specific issues and technical/accessibility issues, respectively.

## 4. Discussion

The IPAT module was developed based on parents’ training needs, as expressed by them in four focus groups held in Italy, Spain, Greece, and Turkey, respectively. We used the IPAT module in four group training activities, delivered in eight weekly sessions of 3 h each, with the participation of 62 parents in total, in Greece, Turkey, Spain, and Italy. We used a pre- and post-training study design, in order to preliminarily explore potential efficacy of the IPAT module and training activity on the short-term parental self-perceived quality of life and stress. In addition, we identified factors associated with QoL and stress and with potential efficacy of the intervention. The satisfaction of participants regarding the IPAT module and training was also measured.

In the present study, the lowest baseline quality of life (QoL) score was observed in the environment domain, while the highest was noted in the physical health domain. Our findings diverge from other research outcomes which demonstrated lower scores concerning physical health, emotional health, or social relationship QoL [24,50,51,52]. Nonetheless, comparing these results warrants caution due to methodological differences in sampling and the utilization of varying measures.

We have identified factors influencing baseline domain-specific QoL and total stress. Among the parent-related factors, parental age and the locality of residence (whether urban or rural) appeared significantly linked to parental quality of life (QoL). Advanced parental age correlated with a decline in the quality of life concerning social relationships. Meanwhile, individuals residing in urban areas exhibited higher levels of quality of life in the environmental domain, compared to their rural counterparts. The influence of parental age on quality of life has sporadically surfaced in previous literature. Both age and gender are reported to impact how parents navigate the challenges associated with caring for a child with ASD [53]. Reduction in the quality of life concerning social relationships might indicate a decline in the individual’s capacity and capability in this domain due to age-related factors. The demanding roles of caregiving and child-rearing often result in social isolation for these parents, reducing opportunities for social interaction beyond the confines of their homes, a situation compounded by a lack of necessary social support [50,52,54]. It is possible to consider the cumulative burden over time, where age is seen as a consistent source of stress for this group of parents, especially when personal or social resources are inadequate, leading to increased strain [55].

Diverging from our findings, previous studies in densely populated (DP) and low-density population (LDP) areas did not identify any differences [56,57]. In line with earlier research, we contend that issues regarding service accessibility and/or difficulties in accessing proficient professionals in rural areas might contribute to the disparities noted. Furthermore, as previously outlined, the mere geographical location and potential challenges encountered in LDP areas do not necessarily heighten stress levels or diminish parental QoL, as long as access to necessary services remains equivalent to that in DP areas [56,58].

Quality of life did not differ significantly between mothers and fathers in our study; however, the majority of previous studies identify a greater negative impact on maternal QoL, more often attributed to the primary caregiver role usually undertaken by mothers, resulting in increased burden and, eventually, different ways parents cope with ASD-related challenges [24,50,53,54,59]. In contrast to previous studies, marital status and education level were not linked to either QoL or stress level in our study [54].

In concordance with previous results, unemployed/homemaker participants were found to be significantly more stressed and/or have a lower QoL than employed/freelancers [50,60,61].

We also observed disparities across countries; participants from Turkey had a lower initial physical health QoL, while participants from Spain had a higher environment QoL, and participants from Italy were initially less stressed, as compared to participants from other countries. In line with the findings of previous studies, differences in socioeconomic status, cultural influences, and the resulting availability and access to formal and informal care resources and services could explain the observed disparities in QoL and stress levels across countries in our study [54,62].

Among the child-related factors, we identified that a child’s low level of needs, enrollment in a special education program, and the duration of enrolled participation in such a program were correlated with parental quality of life (QoL). Parents with stronger social relationship QoL were more likely to have their child enrolled in a special education program compared to those with weaker social relationship QoL. However, the availability of service support is not consistently associated with increased parental QoL [50,60,63]. We consider that this particular service support has the potential to ease the burden of caregiving on parents and grant them more time and availability for socializing, thereby enhancing their social support network.

In agreement with previous studies, participants displaying higher psychological QoL were more likely to have a child with fewer needs or to have their child enrolled in a special education program for a longer duration [64,65]. In the present study, information was gathered about the level of needs based on parents’ reports, without employing standardized measures. Nonetheless, it appears that the self-reported level of the child’s needs or functionality aligns relatively well with the outcomes of standardized measures [52,65].

In contrast to previous studies, we did not explicitly evaluate the impact of behaviors that cause concern on parental quality of life or stress [25,50,52,54,66,67,68,69]. Furthermore, we did not investigate the impact of ASD severity, comorbidities, and additional contextual factors on quality of life (QoL) and reported stress, as found in existing literature [11,24,25,50,51,54,70,71,72].

In line with previous findings affirming the negative correlation between stress and QoL, participants experiencing higher stress demonstrated lower environmental QoL in our study [24,50,52,61,63,73].

Our primary study hypothesis was confirmed with respect to QoL improvement but not with respect to the reduction in stress post-training; furthermore, disparities were not significant across countries, despite the differences observed. Participants’ QoL improved significantly in the environment domain, while they displayed higher average scores post-training in all quality of life (QoL) domains, and 38–57% of them improved their scores. Within countries, only participants from Turkey significantly improved their QoL in all domains post-training. Our findings are similar to those from previous studies, using parent-focused, often hybrid Parent Education and Training (PET) programs, either face-to-face or in telehealth, identifying an improvement in one or more domains of parental QoL post-training [44,74,75,76]. Further, in line with previous research that highlights health-promoting activities, spending time outdoors, and adopting a healthy lifestyle as major contributing factors to quality of life, our study provides evidence that participants had more opportunities for leisure activities, enjoyed life more, and were more satisfied with their sleep after training [60]. However, other studies did not find a significant improvement in QoL post-training, or improvement was identified only during follow-up evaluations [77,78,79]. In either case, results should be interpreted with caution, given the methodological disparities observed across studies, in particular with respect to the different measures used to assess QoL, the existence of a control group, and the type of interventions applied: whether they are parent-focused or parent-mediated, with the latter contingent on the parents’ ability to implement the acquired skills.

The overall stress score did not show a significant reduction, remaining within the initial moderate stress level, although almost half of the participants (48%) had decreased their stress score post-training. Within countries, results varied considerably; stress was not significantly reduced in participants from Greece and Spain, whereas it was not significantly increased in participants from Italy and significantly increased in participants from Turkey. Several previous studies show a reduction in stress post-intervention, while stress levels remain unmodified in others [27,28,74,75]. Certain aspects need consideration when interpreting this outcome; lack of statistical evidence demonstrating a decrease in parental stress post-intervention or persistence of high levels of parental stress, despite the effectiveness of parental training on various other outcomes, has been reported in previous studies [40,79,80,81,82,83]. It is crucial to recognize that reported positive changes might not always translate into statistically significant results. There is also concern regarding potential publication bias due to a high proportion of studies reporting only positive outcomes [45]. Mental health effects have been noted to strengthen over time post-intervention, while parental stress levels have sometimes been observed to rise. Studies have shown that immediately after training, effects might be non-significant or weaker, similar to what was observed in our study. However, significant results were identified during longer-term follow-ups, at least two months after, a factor not accounted for in our study’s design [77,84]. The participants of the present study experienced only moderate, not severe, levels of stress. Yet, previous findings have suggested that poorer mental health could predict better outcomes after an intervention [85].

We have identified one parent-related factor—initially low physical health QoL—and one child-related factor—the number of years the child had spent in a special education program—that might influence the efficacy of the intervention, in terms of post-training improvement of the quality of life (QoL). Parents who experienced an improvement in physical health QoL post-training were more likely to have had a lower physical health QoL at the start compared to those whose scores did not improve. Additionally, parents who had a higher psychological QoL post-training were more likely to have a child who spent more years in a special education program. In essence, the intervention appears to be more beneficial to participants who initially had lower QoL in the physical domain and to those whose children had been in a special education program for a longer period. Previous studies have associated increased effectiveness of mental health interventions with lower initial scores [85]. We have previously detailed the link between higher initial QoL in the psychological domain and the duration of a child’s enrollment in a special education program. Similarly, it could be considered that a longer duration of a child’s enrollment in a special education program allowed parents to gain more from the psychoeducation program, enabling them to better experience the optimal effects of the training conditions.

Although the majority of parents in our study had received their child’s autism diagnosis four or more years before engaging in the IPAT module training activity (66%), their overall satisfaction was notably high, scoring an average of 8.5 on a 1–10 scale. Earlier studies have also reported that participating parents expressed high satisfaction, which correlated with stress reduction, improvements in quality of life, and psychological well-being [50,51].

Parents with higher satisfaction scores were likely to have initially scored lower in the social relationship domain of quality of life (QoL). Participants with lower QoL in the social relationship domain likely placed a high value on expanding their network through group training. They probably found significance in regularly socializing and receiving support from peer parents, as previously mentioned [50,86]. Interacting with peers, especially through social support and validation provided by other parents, has been explicitly identified as a crucial factor leading to improved psychological well-being for parents with a child on the autism spectrum [87]. Conversely, parents who had participated in counselling or psychotherapy programs, either currently or in the past, expressed lower levels of satisfaction. This finding may indicate a reduced relevance or perceived usefulness of the IPAT module’s psychoeducation program for this specific group.

We conducted a study to explore the potential positive impact of the IPAT module training on the quality of life and stress levels experienced by parents of children with autism in four countries: Greece, Italy, Spain, and Turkey. One of the notable strengths of this research is the comprehensive consideration of parents’ expressed needs in a cross-cultural context. Previous studies have recommended evaluating the effects of parental training in the field of autism within a cross-cultural environment [33,34,45]. In our case, this cross-cultural approach coincided with the multi-country aspect, without impinging upon diverse social or ethnic groups within the same country.

Our approach involved incorporating country-specific content tailored to address the availability and accessibility of services and resources, advocacy, rights and legal provisions, family and social support, as well as additional cultural considerations. This practice is strongly advocated in the IPAT module guide for any prospective usage. Although parental stress remained unmodified, there was evidence that QoL of participants improved significantly in the environment domain. Therefore, the present exploratory study provided more insights in line with previous findings that hybrid PET programs, despite not exclusively focusing on teaching stress management skills or techniques to enhance mental health, such as the third wave of CBT, mindfulness, or humanistic-informed interventions, acknowledged as particularly effective for stress management, can positively impact parental QoL and psychological well-being [27,58,76]. The IPAT module includes two of the three practice components associated with improving parental psychological well-being, as previously reported [87]: support provided by peers and the provision of knowledge concerning ASD. Social support offered by peer parents in a group training scenario encompasses informal networking, reducing feelings of isolation and helplessness, and providing valuable validation of experiences and feedback, although findings regarding the impact of group versus individual training have also been considered contradictory [77]. The provision of accurate information and gaining knowledge about ASD has been reported to aid parents in comprehending how individuals with ASD, including their child, experience the world and in understanding their behavioral challenges. This understanding can lead to reduced stress and anxiety and improved well-being [27,74,87]. Furthermore, we have identified factors related to both the parent and the child that mediate the preliminary evidence of post-training improvement in QoL, aligning with previous recommendations in the literature [50]. Lastly, our results may be used to increase accessibility to parental training and support in the autism field and further promote their provision in a telehealth environment, as previously reported [58,76].

There are several limitations within the present study. Firstly, the absence of a control group in our study design stands as the most significant limitation. However, one should consider that inclusion of a control group may intentionally lead to neglecting unmet mental health needs among this particularly distressed group of caregivers, or that waitlist controls would lead to delaying meeting their needs or potentially to higher drop out among them or the introduction of potential selection bias if controls are selected among parents who refuse to participate in support or training activities; a different training mode control group would, however, be a plausible alternative [77]. Furthermore, the relatively small size of our sample, particularly at the national level, may potentially limit the power of our study and increase the likelihood of a type β error. This limitation prevented the identification of effect mediators at the country level.

As for the sample of the present study, this showed enhanced homogeneity concerning the time from diagnosis. Only a small minority of participants had recently received a diagnosis for their child, meaning the impact and satisfaction were not evaluated for parents of newly diagnosed children with autism.

The present study did not include a longer-term follow-up assessment, potentially limiting the identification of stress reduction, as previously observed. However, a larger sample size, the inclusion of a control group, and an extended follow-up duration were impractical within the framework and resources available in the Erasmus+ co-funded IPAT Project. Moreover, our study design did not explicitly address the issue of the child’s expression of behaviors causing concern as a potential factor influencing the post-training quality of life (QoL) and stress. Another issue is that our use of a self-referral convenience sample might not adequately represent all parents of children with autism. Notably, our sample had limited participation from fathers, potentially impacting the representation of the effects of psychoeducation utilizing the IPAT module on fathers’ QoL and stress, despite no identified gender-related differences. Further, we do not report any implementation fidelity results, although some data have been collected to optimize prospect implementation and delivery modes, as included in the IPAT module guide. Lastly, the dropout is significantly high in our study, surpassing the generally acceptable proportion of under 20% [28]; approximately half of the parents (52%) participated in nearly all meetings, while about a third took part in roughly half of the meetings. Though inconclusive, it is acknowledged that interventions focusing on personal growth and psychological well-being tend to experience higher dropout rates than those providing immediate relief from mental health symptoms or stress reduction, which is not the primary objective of our psychoeducational intervention [77].

Increased dropout poses potential issues concerning intervention fidelity, participant engagement, program reach, social relevance, and validity [52]. However, among the twenty-seven parents who left the study early and provided feedback, only one mentioned not benefiting from the intervention, while nineteen cited ‘lack of time and other responsibilities’ as their reason. Prior research has noted the substantial barrier of lack of childcare, hindering participation in training or support activities for parents facing considerable distress due to heightened caregiving demands [86,87,88]. During the initial phases of the IPAT project, discussions with parents underscored the necessity for childcare support during training sessions. However, this specific need remained unfulfilled within the framework of the IPAT Erasmus+ project.

## 5. Conclusions

Despite its limitations, the present exploratory study provided preliminary evidence about the potential positive impact of the psychoeducational IPAT module training program on the QoL of parents of children with autism in a cross-cultural setting, even after receiving their child’s autism diagnosis for a considerable period. Initial moderate stress levels persisted, while parents expressed their high levels of satisfaction about this training program. Utilizing the IPAT module appeared to be more advantageous for parents with lower initial quality of life (QoL) and those whose child had been enrolled in a special education program for an extended duration. At the same time, it was more valued by parents experiencing lower QoL in the domain of social relationships. Carrying out larger-scale studies with extensive sample sizes, including control groups and longer follow-up periods, holds the potential for supporting the preliminary evidence provided by this study, identifying additional moderators, mediators, or effects not currently evident. In addition, they will potentially allow disentangling the contribution of discrete parts of the IPAT module training program, such as the group sessions, provision of knowledge with respect to ASD, and development of specific skills. Future studies need to proactively address barriers to parental participation, particularly in ensuring childcare during training sessions and engaging fathers in the participation process.

The IPAT module training program was developed within the framework of the Erasmus+ co-funded IPAT project, which operated in a cross-cultural context across multiple countries. The program was developed based on the expressed needs of parents at the project’s inception. The IPAT module is freely accessible for non-commercial use by experienced professionals in autism care service provision. It can be administered, shared, specifically tailored to suit children’s age, level of requirements, or cultural context, and/or translated, under the terms of the Creative Commons License, available at ipatproject.eu.

## Figures and Tables

**Table 1:** Demographics and characteristics of participating parents, by country.

**Characteristics**	**Total**	**Greece**	**Spain**	**Italy**	**Turkey**	***p-Value***
N (%)	62 (100.0)	18 (29.0)	18 (29.0)	11 (17.7)	15 (24.3)	
Gender—Parents						0.208 ^a^
Male	8 (13.1)	3 (16.3)	2 (11.8)	3 (27.3)	0 (0.0)	
Female	53 (86.9)	15 (83.3)	15 (88.2)	8 (72.7)	15 (100)	
Age—Parents						0.024 ^b^
Mean (SD) *	42.8 (5.8)	41.1 (7.3)	46.2 (4.5)	43.5 (3.4)	40.8 (5.0)	
Gender—Child						0.116 ^a^
Male	48 (77.4)	15 (83.3)	12 (66.7)	11 (100)	10 (66.7)	
Female	14 (22.6)	3 (16.7)	6 (33.3)	0 (0.0)	5 (33.3)	
Age—Child						0.002 ^b^
Mean (SD)	9.2 (5.3)	6.8 (5.1)	10.6 (6.0)	6.6 (3.2)	12.5 (3.7)	
Diagnosis						<0.001 ^a^
C.A.	36 (58.1)	8 (44.4)	11 (61.1)	3 (27.3)	14 (93.3)	
Autism	6 (9.7)	0 (0.0)	5 (27.8)	0 (0.00)	1 (6.7)	
A.S.	13 (21.0)	10 (55.6)	2 (11.1)	1 (9.1)	0 (0.0)	
Other	7 (11.2)	0 (0.0)	0 (0.0)	7 (63.6)	0 (0.0)	
Number of years since diagnosis						0.013 ^a^
≤1 years	1 (1.6)	0 (0.0)	1 (5.6)	0 (0.0)	0 (0.0)	
2–3 years	21 (34.4)	10 (58.8)	6 (33.3)	5 (45.5)	0 (0.0)	
≥4 years	39 (63.9)	7 (41.2)	11 (61.1)	6 (54.5)	15 (100.0)	
Level of needs—Child						0.183 ^a^
Low	23 (40.4)	9 (60.0)	6 (35.3)	6 (60.0)	2 (13.3)	
Moderate	22 (38.6)	3 (20.0)	8 (47.1)	4 (40.0)	7 (46.7)	
High	12 (21.0)	3 (20.0)	3 (17.6)	0 (0.0)	6 (40.0)	
Participation counselling/psychotherapy						0.009 ^a^
Yes	16 (26.2)	10 (55.6)	2 (11.8)	2 (18.2)	2 (13.3)	
No	45 (73.8)	8 (44.4)	15 (88.2)	9 (81.8)	13 (86.7)	
Number of meetings attended						0.002 ^a^
1–3	9 (16.4)	4 (26.7)	4 (28.6)	0 (0.0)	0 (0.0)	
4–6	17 (30.9)	5 (33.3)	5 (35.7)	6 (54.5)	2 (13.3)	
7–8	29 (52.7)	6 (40.0)	5 (35.7)	5 (45.5)	13 (86.7)	
Mean (SD) *	5.9 (2.2)	4.6 (2.7)	5.2 (2.5)	6.5 (1.2)	7.2 (0.9)	

Values are presented as absolute (N) and relative frequencies (%), unless stated otherwise. ^a^ Chi-square test, ^b^ Student’s *t*-test, * SD = Standard deviation; C.A. = Childhood Autism; A.S =Asperger’ s Syndrome.

**Table 2:** Quality of life by domain and perceived stress scores, by administration and country.

QoL	Total	Grece	Spain	Italy	Turkey
QoL—Satisfaction with quality of life					
Pre-Training	3.6 (0.7)	4.1 (0.5)	3.7 (0.5)	3.5 (0.7)	2.9 (0.7)
Post-Training	3.7 (0.7)	4.0 (0.4)	3.7 (0.8)	3.5 (0.5)	3.5 (0.9)
p-value *	0.105	0.583	0.719	1.000	0.045
QoL—Satisfaction with health					
Pre-Training	3.5 (0.9)	3.7 (0.6)	3.6 (1.0)	3.7 (0.8)	3.1 (1.2)
Post-Training	3.6 (0.9)	3.4 (0.9)	3.2 (1.0)	3.7 (0.6)	3.9 (0.6)
p-value *	0.799	0.104	0.138	1,000	0.034
QoL—Physical Health					
Pre-Training	62.8 (16.5)	67.9 (11.4)	70.2 (14.1)	67.9 (14.9)	47.4 (14.2)
Post-Training	67.5 (15.1)	65.4 (18.2)	68.1 (16.0)	67.2 (15.3)	68.8 (12.2)
p-value *	0.058	0.474	0.480	0.813	0.001
QoL—Psychological					
Pre-Training	61.3 (15.7)	66.0 (6.1)	66.4 (13.3)	53.4 (21.1)	57.8 (17.4)
Post-Training	64.5 (15.6)	63.1 (15.6)	70.8 (12.3)	53.8 (18.7)	67.2 (13.3)
p-value *	0.117	0.549	0.092	0.882	0.081
QoL—Social relationships					
Pre-Training	59.6 (21.2)	65.5 (14.6)	60.1 (18.8)	64.4 (23.3)	50.0 (25.2)
Post-Training	65.0 (19.7)	61.9 (22.8)	58.9 (13.7)	65.2 (20.0)	73.3 (20.2)
p-value *	0.103	0.362	0.770	0.821	0.021
QoL—Environment					
Pre-Training	56.1 (16,4)	55.2 (9.7)	65.6 (13.8)	54.8 (17.2)	48.8 (19.2)
Post Training	62.4 (16.1)	53.9 (13.2)	67.2 (11.4)	53.4 (12.5)	71.5 (18.4)
p-value *	0.011	0.508	0.530	0.588	0.002
Total perceived Stress score					
Pre-Training	15.1 (4.8)	16.5 (3.6)	15.0 (5.6)	11.3 (4.2)	16.5 (4.2)
Post-Training	14.4 (6.7)	12.8 (8.3)	12.7 (6.9)	12.8 (3.3)	19.7 (3.2)
p-value *	0.453	0.073	0.227	0.222	0.048

Values are presented as mean and standard deviation, unless stated otherwise. * Paired samples *t*-test.

**Table 3:** Participants presenting with improved quality of life score, by domain and reduced total perceived stress score, post-training, by country.

QoL	Total	Greece	Spain	Italy	Turkey
QoL—Psychical health	25 (46.3)	4 (22)	5 (30)	5 (45)	11 (73)
QoL—Psychological	31 (57)	6 (33)	8 (44)	7 (64)	10 (67)
QoL—Social Relationships	21 (38)	5 (28)	2 (11)	4 (36)	10 (67)
QoL—Environment	26 (49.1)	3 (17)	6 (33)	5 (45)	12 (80)
Total perceived Stress score	25 (48.1)	5 (30)	5 (30)	6 (54)	9 (60)

Values are presented as absolute (*n*) and relative (%) frequencies, unless stated otherwise.

**Table 4:** Satisfaction with the IPAT module and training activity.

Participants’ Satisfaction with Respect to	Mean (SD)	Median (IR)
Training overall	8.5 (1.4) ^a^	9.0 (2.0) ^b^
Lectures	8.4 (1.7) ^a^	9.0 (2.3) ^b^
Experiential/interactive activities	8.5 (1.6) ^a^	9.0 (2.0) ^b^
Question answering and discussion	8.6 (1.5) ^a^	9.0 (2.0) ^b^
Moderators	9.0 (1.4) ^a^	9.0 (2.0) ^b^
Fulfillment of expectations	8.4 (1.7) ^a^	9.0 (2.0) ^b^

^a^ Mean value (standard deviation); ^b^ median value (interquartile range).

## Data Availability

The data presented in this study are available on request from the corresponding author. The data are not publicly available due to privacy and ethical considerations; consent was provided by participants so that data would be used by the members of the research team and for the purposes of the study.
